# Drug-loadable Mesoporous Bioactive Glass Nanospheres: Biodistribution, Clearance, BRL Cellular Location and Systemic Risk Assessment via ^45^Ca Labelling and Histological Analysis

**DOI:** 10.1038/srep33443

**Published:** 2016-09-15

**Authors:** Baiyan Sui, Gaoren Zhong, Jiao Sun

**Affiliations:** 1 Shanghai Biomaterials Research & Testing Center, Shanghai Key Laboratory of Stomatology, Ninth People’s Hospital, Shanghai Jiaotong University School of Medicine, Shanghai 200023, China; 2 School of Pharmacy, Fudan University, Shanghai 201203, China

## Abstract

Mesoporous bioactive glass (MBG) nanospheres with excellent drug loading property have attracted significant attention in the field of nano-medicine. However, systemic metabolism and biosafety of MBG nanospheres which are crucial issues for clinical application are yet to be fully understood. Isotope quantitative tracing combined with biochemical parameters and histopatological changes were used to analyze biodistribution, excretion path and the effect on metabolism and major organs, and then we focused on the hepatocellular location and damaging effect of MBG. The results indicated MBG possessed a longer residence time in blood. After being cleared from circulation, nanospheres were mainly distributed in the liver and were slightly internalized in the form of exogenous phagosome by hepatocyte, whereby more than 96% of nanospheres were located in the cytoplasm (nearly no nuclear involvement). A little MBG was transferred into the mitochondria, but did not cause ROS reaction. Furthermore, no abnormal metabolism and histopathological changes was observed. The accumulation of MBG nanospheres in various organs were excreted mainly through feces. This study revealed comprehensively the systemic metabolism of drug-loadable MBG nanospheres and showed nanospheres have no obvious biological risk, which provides a scientific basis for developing MBG nanospheres as a new drug delivery in clinical application.

In recent years, tremendous efforts have been devoted to the development of mesoporous silica-matrix nanoparticles which is used in drug delivery field^1^,^2^. Thereinto, mesoporous bioactive glass (MBG) nanospheres have a composition SiO_2_-CaO-P_2_O_5_ with the mesoporous structure, which possess high specific surface area, large pore volume, tunable mesoporous size, bioactivity and a certain degree of degradation. These characters of MBG, is different from mesoporous silica nanoparticles that are widely used as a drug delivery, have been considered as a potential new candidate for drug controlled-release delivery^3^,^4^. Although it has been reported that MBG nanospheres have the advantages of high loading efficiency and sustained release of drug^5^, it is not known whether MBG nanospheres can be used as a delivery *in vivo*. There are two critical problems that must be solved, which include the metabolism process and biological safety of MBG *in vivo*. However, nowadays the research on distribution *in vivo*, residence time in blood, excretion pathway, the effect on physiological functions of major organs of MBG nanospheres still have a gap. Therefore, to assess the viability of drug-loadable MBG nanospheres in the clinical setting, it is necessary to explicit systemic metabolism processes and the risks of using MBG nanospheres.

MBG nanospheres are mainly composed of silica and calcium^6^. At present, to detect the distribution of silica matrix nanoparticles *in vivo*, inductively coupled plasma-optical emission spectroscopy and inductively coupled plasma-mass spectrometry are used to determine the silica content^2^,^7^,^8^. These methods were used to acquire the quantitative data, but most of them have some problems, for example, the sensitivity of detection was reduced due to diluting samples before testing, and the data was easily subjected to interference by allochthonous impurities during the processing of samples or inherent elements in the body. Another tracing method, which was set up by radiolabelling mesoporous silica nanoparticles using ^125^I or ^64^Cu^9^,^10^, could effectively avoid the interference of autologous elements. However, this method requires an extra and complex labeling process, and the physicochemical property and *in vivo* distribution of nanoparticles can undergo changes due to the chemical bonding between radionuclide and nanoparticles. Therefore, so as to establish a labeling technique, which has the advantages of being relative simple, accurate and not impact the nanoparticles’ physicochemical properties, is one of the important aspects that was considered in our study. The presence of calcium in MBG nanospheres makes it possible to trace MBG nanospheres *in situ* via ^45^Ca radionuclide labeling. ^45^Ca, which has a suitable half-life, can be used to monitor the distribution *in vivo* with long time limitation and specificity.

A lot of research have shown that most drug nano-delivery systems accumulate in the liver^11^,^12^, which may be due to the xenobiotic metabolism function of the liver, this property not only interferes with the purpose of delivery for systemic administration, but also may have adverse effects on hepatocellular or physiological function. Liver is an important metabolic organ, which acts in the catabolism of poisonous substances and participates in glycometabolism, lipometabolism and protein metabolism, and is the root of demichomeostasis. Furthermore, it will cause an inflammatory infiltrate and even hepatocyte necrosis at the portal triads if the accumulation of nanoparticles cannot be excreted effectively^13^. Therefore, it is especially important to evaluate the particle-induced hepatocyte interactions including intracellular localization and cellular function alteration, and pay attention to metabolism and histopathology.

In the present study, MBG nanospheres was synthesized and labeled *in situ* by introducing radionuclide ^45^Ca. So as to better understand the regular pattern of MBG nanospheres systemic metabolism, we studied their residence time in blood, distribution and accumulation in various organs, excretion pathway, and further studied hepatocellular intracellular locations and the effects on mitochondrial function. Meanwhile, from the perspective of biological safety, the impact of MBG nanospheres on physiological function and major organs was systemically assessed by biochemical analysis and histopathological examination. Our study provide critical scientific basis for clinical application of drug-loadable MBG nanospheres.

## Results

### Characterization of MBG nanospheres

**Figure 1:**
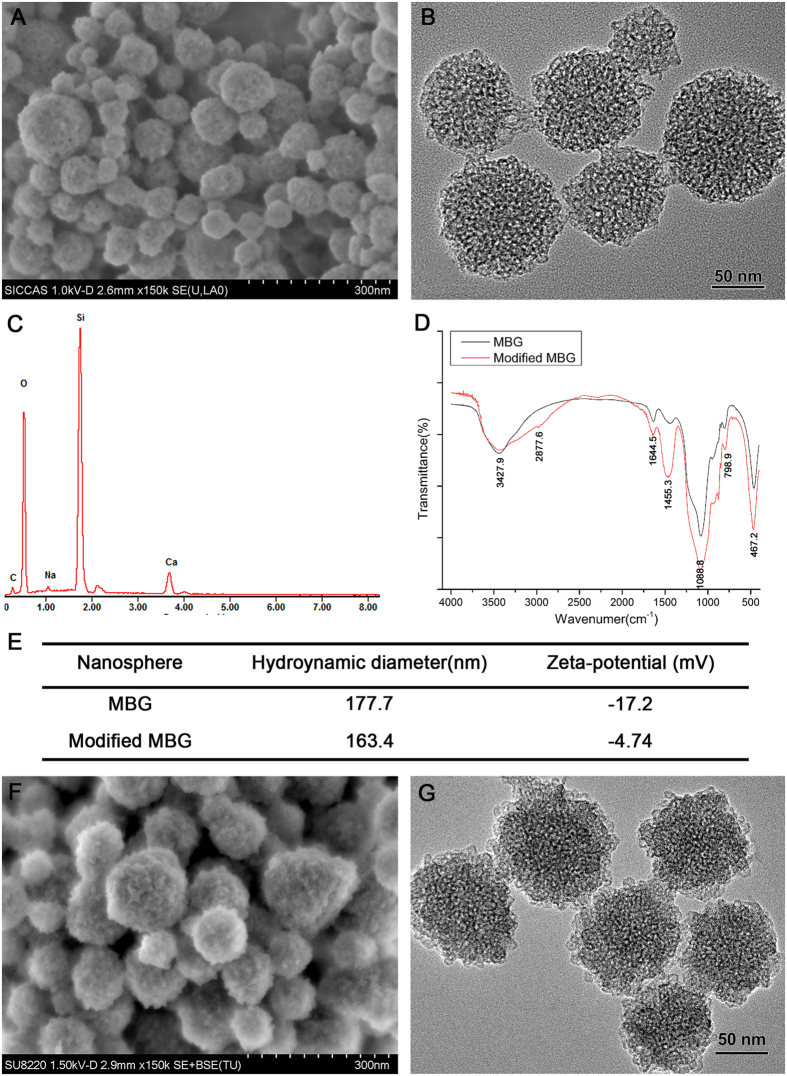
Characterization of MBG nanospheres. **(A–C)** SEM, TEM and EDS analysis of MBG nanospheres; **(D)** FT-IR spectra of MBG and modified MBG nanospheres; **(E)** Nanospheres hydronamic diameter and zeta potential; and **(F,G)** SEM and TEM analysis of modified MBG nanospheres.

The spherical morphology of MBG nanospheres was confirmed by SEM (Fig. 1A). TEM results showed that MBG nanospheres possessed a three-dimensional wormhole-like mesostructure, and the size of nanospheres was in the range of 50–100 nm (Fig. 1B). EDS analysis revealed that calcium and silicon elements were the main component of MBG nanospheres (Fig. 1C). MBG nanospheres were modified with APTES, for making them with multi-function to adapt to clinical demands. It has been reported that the introduction of functional groups to nanoparticles by APTES could facilitate the coupling of nanoparticles with drugs, fluorescein, and decrease drug burst release of drug carriers^14^,^15^. Our FTIR analysis (Fig. 1D) showed the FT-IR spectra peaks at the bands of unmodified MBG attributed to Si-O-Si bending (467.2 cm^−1^), Si-O-Si symmetric stretching (798.9 cm^−1^), external Si-OH groups (1088.8 cm^−1^), water molecules retained by siliceous materials (1644.5 cm^−1^) and -OH stretching (3427.9 cm^−1^)^16^. After modification with APTES, MBG still retained its silicon matrix structure. The band at 1455.3 cm^−1^ presented a more intense vibration formed by the protonated amine groups (−NH^3+^), which may be related to the aqueous phase of amino adsorption or neighboring silanol groups^17^, and this phenomenon demonstrated that MBG nanospheres were successfully grafted an -NH_2_ group. Meanwhile, the result of zeta potential showed that the modified MBG nanospheres potential was raised from the original −17.2 mV to −4.74 mV (Fig. 1E), which belonged to neutral charge range (within ± 10 mV)^18^. The morphology and size of modified MBG nanospheres show no significant change compared to that of unmodified MBG nanospheres (Fig. 1F,G).

### Identification of ^45^Ca-MBG and degradation of MBG nanospheres

**Figure 2:**
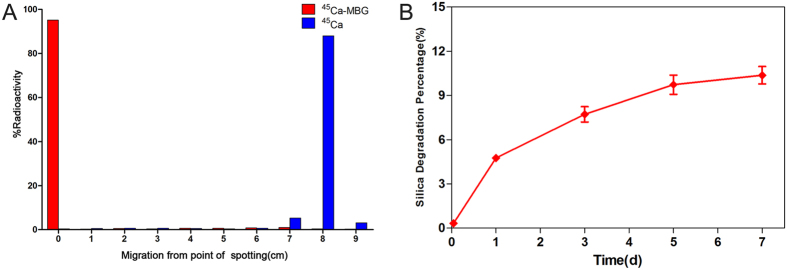
The identification of ^45^Ca-MBG nanospheres **(A)** and the degradation of MBG nanospheres **(B)**.

After radiolabeling and synthesizing, the ^45^Ca-MBG nanospheres obtained were identified by the ITLC-SG. The result of ITLC-SG showed that radionuclide ^45^CaCl_2_ moved toward the solvent front (RF = 8), but almost all of ^45^Ca-MBG nanospheres remained at the point of spotting (>95%, RF = 0) (Fig. 2A). It suggested that the ^45^Ca-MBG nanospheres were stable and had high purity. As shown as in Fig. 2B, the MBG nanospheres degraded slightly in mSBF, the accumulated released amount of Si was only 10.37 ± 0.59% during 7 days, and the release curve had flatten out gradually. It demonstrated that there was no obvious degradation of MBG nanospheres.

### Quantitative and qualitative distribution of ^45^Ca-MBG nanospheres

**Figure 3:**
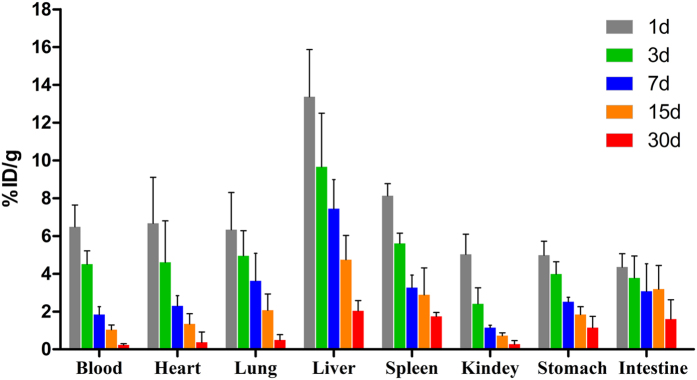
LSC quantitative measurement of tissue distribution of ^45^Ca-MBG nanospheres.

The residence time in blood and distribution were evaluated by LSC within 30 days after intravenous injection with ^45^Ca-MBG nanospheres. The blood retention of MBG nanospheres was 6.49 ± 1.15% ID/g at day 1, and then gradually declined over the next four time points (Fig. 3). Meanwhile, ^45^Ca-MBG nanospheres were mainly distributed in the major organs including the heart, lung, liver, spleen, stomach, intestine and kidney, and accumulated primarily in liver. The accumulation amounts of nanospheres in various organs peaked at day 1, and gradually decreased to less than 2% ID/g after 30 d, except that of MBG nanospheres in the liver.

**Figure 4:**
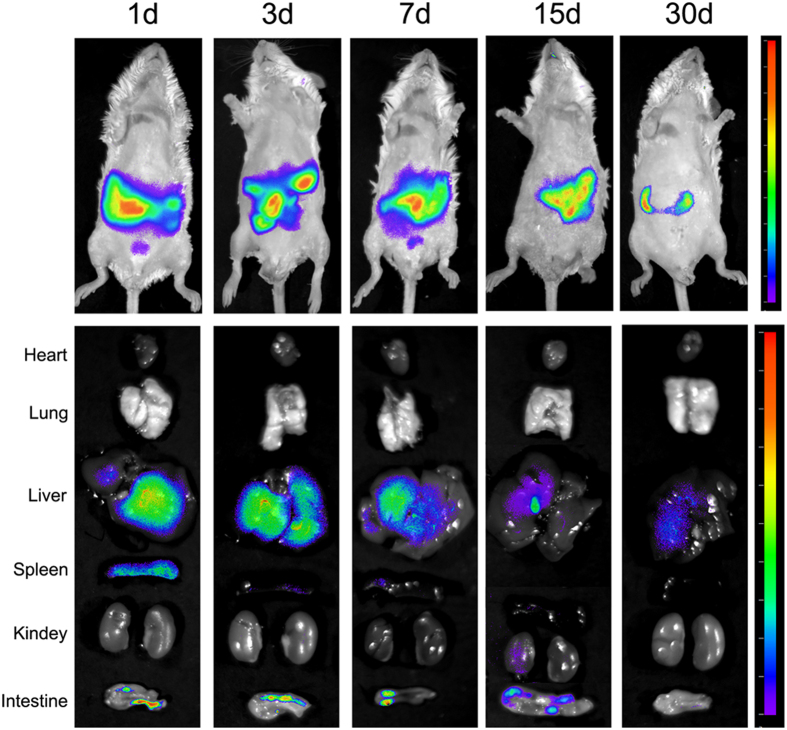
Qualitative tissue distribution of MBG nanospheres. Animals and harvested tissues were imaged using an *ex vivo* fluorescent imaging system.

The qualitative tissue distribution was imaged by the fluorescence imaging system post-injection with Cy7-MBG nanospheres. As can be seen from Fig. 4, fluorescence signal was clearly visualized in abdomen of mice, and the intensity of signal decreased with time. Besides, the result of the *ex vivo* evaluation of major organs, liver and intestines exhibited higher fluorescent signal than those of other organs. The results of qualitative and quantitative tissue distribution have confirmed that MBG nanospheres were mainly distributed in liver.

### Ultrastructure distribution of MBG nanospheres in liver

**Figure 5:**
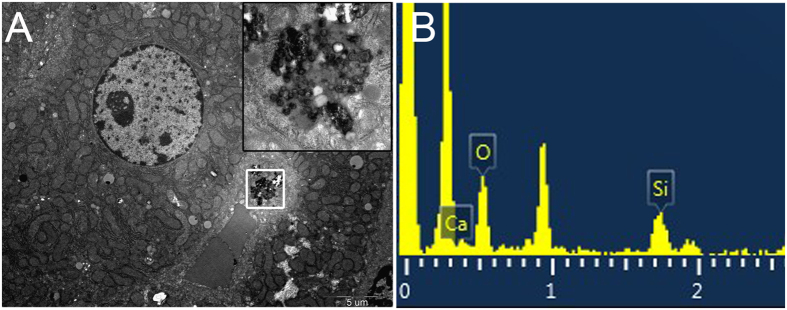
TEM images **(A)** and EDS **(B)** analysis of liver after injection with MBG nanospheres at 7 d. The white pane denoted phagosome containing nanospheres, and were magnified on the right corner of the same image and analyzed by EDS.

TEM-EDS was used to get more details about the cellular distribution of MBG nanospheres in the liver. Our research discovered that some exogenous phagosomes appeared in hepatocytes located in the liver lining the walls of the sinusoids at 7 days post-injection of MBG nanospheres (Fig. 5A). Elemental analysis indicated that granular material in phagosomes contained calcium and silicon (Fig. 5B), which correspond to the major component of MBG nanospheres (Fig. 1C). It indicated that the exogenous phagosome were MBG nanospheres. Until the 30^th^ day, there were nearly no MBG nanospheres involved with cells in liver under TEM observation (Suppl. Fig. 1).

### Cell viability of MBG nanospheres

**Figure 6:**
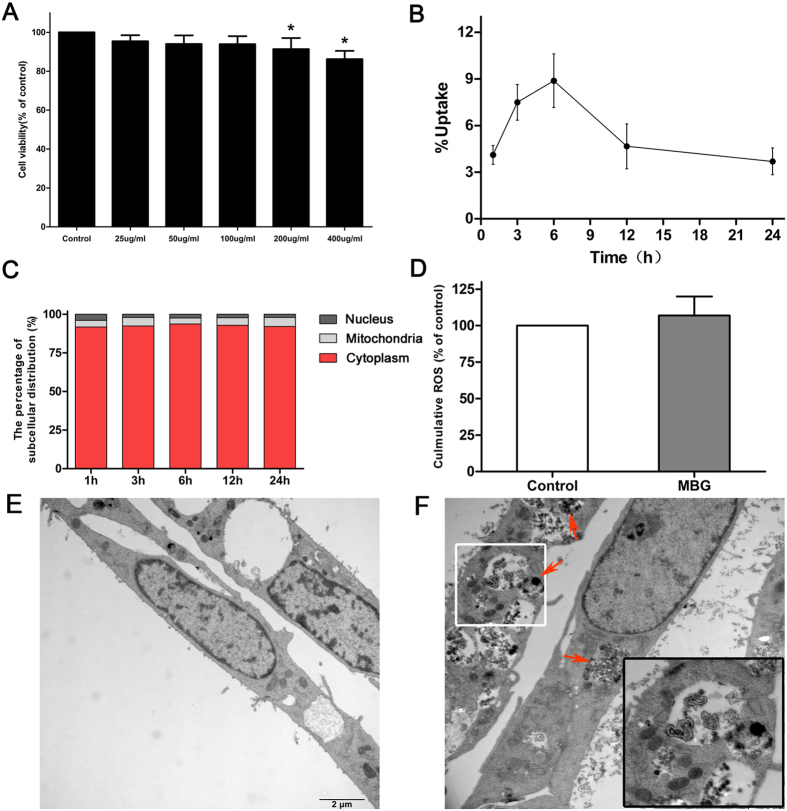
(**A**) Cytotoxicity of MBG nanospheres to BRL cells. The results were presented as the mean ± SD. *p < 0.05 vs. control; **(B)** Cellular uptake of ^45^Ca-MBG nanospheres was measured at each time point; **(C)** Subcellular distribution of ^45^Ca-MBG nanospheres, data are expressed as the mean percentage of total cellular radioactivity; **(D)** ROS levels of cells incubated with of MBG nanospheres for 24 hours; **(E)** TEM micrographs of cells without any treatment; and **(F)** TEM micrographs of cells exposed for 24 h to MBG nanospheres. Red arrows denote nanospheres. The white pane denoted lysosomes surrounding nanospheres, and were magnified on the right corner of the same image.

To assess the biological effect of MBG nanospheres on BRL cells, cell viability was determined after exposing MBG nanospheres at different concentrations for 24 h. As shown in Fig. 6A, a dose-dependent inhibition of cell viability was observed after exposure to MBG nanospheres. Because the subsequent experiments required functioning normally and metabolizing actively cells, an appropriate dose (100 μg/mL) that did not significantly affect the viability of BRL cells (P > 0.05) was applied in follow-up tests.

### Uptake and location of MBG nanospheres by BRL cells

To comprehend the interactions of MBG nanospheres with BRL cells, the uptake of MBG nanospheres by BRL cells was measured with LSC. The results showed that the intracellular amount of MBG nanospheres reached a maximum of 8.88 ± 1.73% at 6 h after the exposure of materials, and then declined gradually with time (Fig. 6B). Furthermore, radioactive isotope tracer combined with TEM was used to analyze the distribution and content of MBG nanospheres in the nucleus, mitochondria and cytoplasm during 24 hours. As shown in Fig. 6C, more than 96% of the internalized MBG nanospheres remained in the cytoplasm and 3.79–5.77% of the total MBG nanospheres were involved in the mitochondria, but nearly none was in the nucleus. Similar results were depicted in Fig. 6F, zooming in on the cytoplasm of BRL cells, we found that the agglomerated nanospheres were enriched in the cytoplasm, but rarely in the nucleus. These results indicated a small part of MBG nanospheres could enter into BRL cells, and were mainly located in the cytoplasm.

### Oxidative stress induced by MBG nanospheres in BRL cells

To investigate the induction of oxidative stress in BRL cells by MBG nanospheres, ROS production was monitored by the fluorescence intensity of dichlorofluorescein. As shown in Fig. 6D, the level of intracellular ROS generation in BRL cells did not significantly enhanced (P > 0.05) after exposing 100 μg/mL of MBG nanospheres for 24 h. This result suggested that 100 μg/mL MBG nanosphere did not stimulate BRL cells to generate ROS.

### Excretion of MBG nanospheres through urine and feces

**Figure 7:**
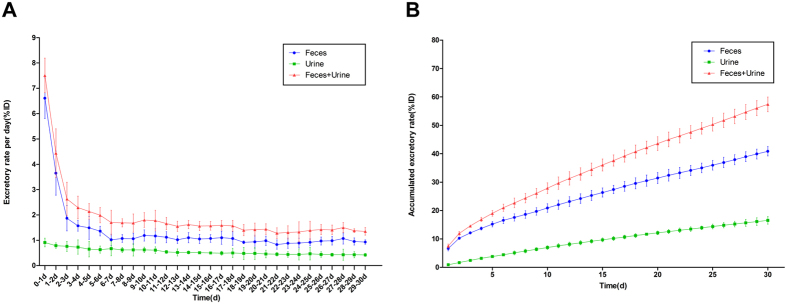
Excretory rate of MBG nanosphers through feces and urine. The excretion of MBG nanospheres was evaluated in the form of excretory rate per day **(A)** and accumulated excretory rate **(B)**.

LSC was used to measure the content of drug-loadable MBG nanospheres in urine and feces per day. The results showed that the excretion rate per day of MBG nanospheres in feces was higher than that of nanospheres in urine (Fig. 7A). Up to 30 days, the accumulated excretion of MBG nanospheres through feces reached 40.85 ± 1.64% ID, while that of urine was only approximately 16.54% ID (Fig. 7B). These results indicate that MBG nanospheres are mainly excreted through feces.

### Clinical biochemistry analysis and histopathological observation

**Figure 8:**
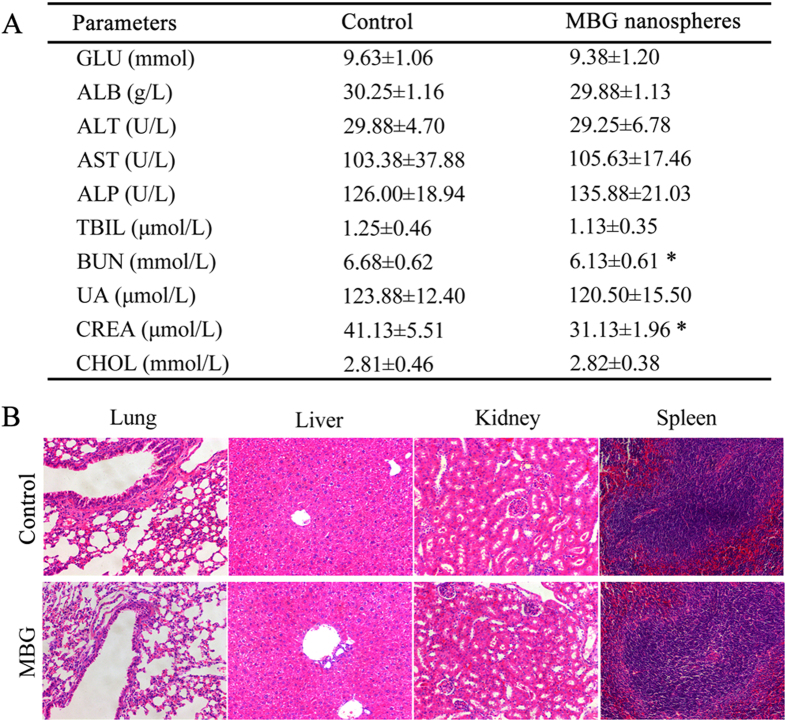
(**A**) Effect of MBG nanospheres on selected clinical chemistry parameters; **(B)** Representative sections from mice tissues after continuous injection with MBG nanospheres. All sections were stained with H&E and observed under a light microscope at 200× magnification.

During the continuous intravenous injection of MBG nanospheres within 7 days, the weights of mice maintained a stable growth (Suppl. Fig. 2). Furthermore, except for BUN and Creat, in the MBG nanospheres group various serum biochemical parameters which represent the body’s metabolism, did not experience a significant increase as compared to the control group (Fig. 8A, P > 0.05). The insignificant decrease in Bun and Creat in the MBG nanospheres group did not represent anything significant clinically speaking. In addition to this, the histological sections of the involved organs including heart, lung, liver, spleen and kidney were no obvious pathological change in major organs after the continuous injection of MBG nanospheres (Fig. 8B and Suppl. Fig. 3).

## Discussion

MBG nanospheres showed excellent drug controlled release, a high loading efficiency of drug reaching up to 90%, and therefore have a promising application in the nano medicine field^5^,^6^. However, systemic metabolism and risk to organism of MBG nanospheres play key roles in application performance and clinical applicability in the future. Therefore, in our study, radionuclide ^45^Ca labeling technique was used to synthesize the quantitative traceable MBG nanospheres *in situ*. The systemic metabolism of MBG nanospheres was examined from three aspects, including the retention in blood, the distribution and the ultimately the excretory pathway. Meanwhile, the possibility of hepatocellular injury, the cellular uptake, location and ROS reaction of MBG nanoshperes internalized by the BRL cells were explored. Finally, we evaluated the biological risk of MBG nanospheres with the help of histopathology and blood biochemical indexes.

To better understand the effects of MBG nanospheres on the systemic metabolism and the potential risks of MBG nanospheres, the establishment of an effective labeling technique is considered as monitoring the process of long-term dynamic changes of MBG nanospheres *in vivo*. Radionuclide labeling has been widely applied in the field of drug, protein and nanoparticles tracing *in vitro* and *in vivo*^19^,^20^,^21^,^22^. Our previous study showed that ^45^Ca radionuclide provided the advantage of labeling MBG scaffolds *in situ* without changing the material structure and properties^23^. Therefore, this study used the advantages of ^45^Ca in labeling and synthesizing MBG nanospheres, which have a roughly uniform size and mesoporous structure (Fig. 1A,B). The stability and high-purity of ^45^Ca-MBG nanospheres as shown in Fig. 2A indicated the labeling technique was suitable for the subsequent tracing research *in vitro* and *in vivo*. Through there were ting degradation of MBG nanospheres (Fig. 2B), as we known, the apatite phase of MBG, which was formed by the Ca^2+^ accumulated and the attracted negatively charged PO_4_^3−^ in SBF^24^, may act as a passivation layer that would inhibit further dissolution of silica and calcium, slow down and even stop the degradation. Therefore, the slight degradation behavior of MBG nanospheres (almost 90% MBG nanospheres were no degradation) will not be the interference to the follow-up testing including the distribution and excretion of MBG nanospheres *in vivo*. In addition to this, to observe visually MBG nanospheres distribution *in vivo*, Cy7 was grafted onto the amino MBG nanospheres. Cy7 exhibited fluorescence in the near infrared region (700–1000 nm), intended for imaging agents, as this kind of fluorescein resulted in a lower background signal and deeper penetration into biometrics^25^, which makes it possible to survey the biodistribution of MBG nanospheres *in vivo* for an extended period.

On the basis of the successful preparation of MBG nanospheres with tracing function, we first focused on the retention of MBG in blood circulation within 30 days. This is because the loading drug’s bioavailability was expected to be prolonged by raising the retention of drug delivery in circulation during the systemic metabolism of drug delivery. To our surprise, the retention of MBG nanospheres (ξ = −4.74 mV) was 6.49 ± 1.15% ID/g at day 1, and still maintained 0.23 ± 0.07% ID/g at day 30 (Fig. 3), which was higher than that of nanoparticles with similar size and amino-modified as we previously report (0.01−0.06% ID/g)^13^,^22^. This phenomenon might be associated with the level of the nanoparticle’s potential, which was one of the critical factors to impact blood retention of nanoparticles^26^. Positive or negative nanoparticles (ξ ≤ −10 mV or ≥10 mV), which easily adsorbed proteins such as IgG and albumin after entering the body, were efficiently opsonized and seceded quickly from the circulation^27^,^28^. It caused the persistence of nanoparticles in blood in an extremely low dose. Compared to non-mesoporous nanoparticles, the high specific surface area of MBG nanospheres, which was assigned by the mesoporous structure, made that the nanospheres in our study was more easily modified with APTES under a similar reaction condition, the potential of MBG nanospheres with APTES modification was increased and closer to neutral. This property might have reduced the adsorption of opsonin-related proteins, and prolonged the delivery’s resistance time in blood. It created the prerequisites for efficient development of drug bioavailability in the future.

We then looked into the question of the destination of MBG nanospheres cleared from blood. The accumulation of MBG nanospheres in liver was higher than that of MBG nanospheres in other organs (Fig. 3). There was a stronger signal in the liver when fluorescence imaging was used, which further supported the above finding (Fig. 4). The relatively high accumulation level in the liver may be explained by two points. On one hand, the material’s size was close to vascular fenestrations in liver^26^, which may benefit the transportation of MBG nanospheres in the liver. On the other hand, liver is an important place not only for metabolism but is also targeted aggregation organ of exogenous foreign body.

Since liver was the major organ where MBG nanospheres accumulated, it was necessary to identify whether MBG got into hepatocytes. Our results revealed that MBG nanospheres were internalized in the form of exogenous phagosome by hepatocytes (Fig. 5). Although the exogenous phagosome had disappeared at 30 day (Suppl. Fig. 1), we had to pay high attention to the existence of MBG nanospheres in the liver cells. This is because hepatocytes are the main part of the liver, they are the liver’s foundation for the metabolism of glucose, protein and lipid, undertake signal transduction, detoxication and homeostasis. Internalization of nanoparticles in the liver cells could potentially cause anomaly in cellular function. The extent of reaction was decided by the intracellular position (nucleus, mitochondria or other organelles) of nanoparticles involvement. If by entering into the nucleus, the nanoparticles could induce cellular apoptosis, and this would have raised undoubtedly the application risk of MBG nanospheres.

To resolve the query, we still adopted the radionuclide quantitative tracer technology, and applied the *in vitro* model. In the normal condition of cell viability, the uptake of MBG nanospheres by BRL cells was analyzed. We then probed into the distribution and content of MBG nanospheres in the nucleus, mitochondria and cytoplasm. To our relief, the most internalized MBG nanospheres parked in the cytoplasm, but nearly none were in the nucleus (Fig. 6C,F). Zooming in on Fig. 6F, the most MBG nanospheres hang out in the cytoplasm in the form of endosome, and some lysosomes were recruited gradually around MBG nanospheres. To our relief, only individual lysosomes began to fuse with the endosome (the color of lysosome will be darkening after engulfing nanoparticles). Thus it was evitable to the cellular damaging effect induced by nanoparticles, for the abundant nanoparticles that sequestered by lysosome could leaded to the obvious cytotoxicity^29^,^30^. The dimension of agglomerated nanospheres was more than 100 nm, which was larger than nuclear pore complex^31^. The results indicated that it was difficult for nanospheres to get into the nucleus, and prompted that MBG nanospheres in hepatocyte could not have a negative effective on the nuclear function.

We were also interested in knowing whether nanospheres transferring into the mitochondria (3.79–5.77%, Fig. 6C) interfere with cellular function. Certain studies have found that nanoparticles could directly damage mitochondria, cause mitochondrial swelling, and then interfere with cellular function^32^. The level of intracellular ROS was an important indicator for evaluating whether cell function was damaged. Excessive production of ROS could activate multiple signaling pathways to induce mitochondrial damage, cell dysfunction and apoptosis^33^. Therefore, the intercellular ROS level in BRL cells that was incubated with MBG nanospheres for 24 hours was determined. The result excluded the possibility that hepatocyte was injured by nanospheres involved in the mitochondria (P > 0.05, Fig. 6D). In these experimental conditions, this series of results suggested microscale internalized MBG nanospheres by hepatocyte mainly located in cytoplasm, although rarely involved with the mitochondria, they did not interfere with cellular function.

The excretion of MBG nanospheres, which was an important aspect of MBG nanosphere’s systemic metabolism, was analyzed by LSC. The results that the accumulated excretion of MBG nanospheres through feces was obviously superior to that of urine (Fig. 7), demonstrated that MBG nanospheres were excreted mainly through feces. The fluorescence imaging *in vitro* (Fig. 4) revealed that a certain intensity of fluorescent signal were observed in intestines, and in the gall bladder at 15 days, which further supported the conclusion above. For larger metabolites, such as MBG nanospheres in our study, the preferred route of excretion is through the fecal matter, via the liver and the bile. Protein binding and phagocytosis by RES plays a critical role in removing the xenobiotic metabolites from the plasma and delivering them to the liver, whereby they are excreted out as feces along with bile^2^. This part result provided a necessary scientific data to understand the systemic metabolism of MBG nanospheres.

For any of the existing clinically approved diagnostic formulations, the most favored route of excretion is still the renal route, as opposed to the hepatobiliary route. This is because hepatobiliary excretion is a slow process, and long-term retention in the RES increases the likelihood of organ toxicity due to the injected nanoparticles^21^. Therefore, it is important to define whether nanoparticles themselves would induce toxicity and dysfunction in the blood and organs, prior to their fecal excretion. For that, we have carried out serum biochemical indexes and histological analysis. To our relief, representative serum biochemical indicators, which were used as biochemical markers for liver and kidney function, were within the normal range, indicating that MBG nanospheres involved various organs did not influence host function. Meanwhile, there were no apparent histopathological abnormalities or lesions for the group treated with MBG nanospheres compared to that of the control group. These results demonstrated that MBG nanospheres possess a favorable biological safety, and the whole-body distribution of MBG nanospheres cannot generate application risk.

## Conclusion

This study has successfully prepared drug-loadable MBG nanospheres with tracing function by *in situ* labeling synthesis of radionuclide ^45^Ca. Through quantitative and qualitative research about systemic metabolism of MBG nanospheres, the results demonstrated that MBG nanospheres have a longer residence time in blood, making it possible to efficiently provide appropriate loading drug’s bioavailability in the future. Then MBG nanospheres that exited from blood circulation were primarily distributed in the liver and were slightly internalized by hepatocyte, more than 96% of which were located in the cytoplasm, but hardly involved the nucleus. The MBG nanospheres transferred into the mitochondria did not cause the cell to generate excessive ROS, and the implication of hepatocellular function had been ruled out. MBG nanospheres distributed in various organs didn’t cause histopathological or biochemical indices abnormality, were excreted mainly through the feces within 30 days, and exhibited good biosecurity. All these findings suggest that our prepared drug-loadable MBG nanospheres can be developed further and be applied as a new drug delivery system.

## Methods

### Materials

Polyvinylpyrrolidone (PVP, K30), cetyltrimethylammonium bromide (CTAB), tetraethyl orthosilicate (TEOS), tetrahydrate calcium nitrate (Ca(NO_3_)_2_·4H_2_O), triethyl phosphate (TEP), (3-Aminopropyl) triethoxysilane (APTES) and dimethyl sulfoxide (DMSO) were purchased from Sigma-Aldrich (St. Louis, MO). Calcium-45 Radionuclide (74 MBq/batch) and Ultima Gold™ were obtained from Perkin Elmer (Massachusetts, USA). Cyanine7 NHS ester was from Lumiprobe (USA). Cell Counting Kit-8 assay (CCK-8) was acquired from Beyotime Biotechnology (Shanghai, China). KeyGen Mitochondria/Nuclei Isolation Kit was from KeyGEN BioTECH (Jiangsu, China). 2′,7′-dichlorofluorescein diacetate (DCFH-DA) was purchased from Applygen Technologies Inc. (Beijing, China). Perchloric acid, hydrogen peroxide, nitric acid, sodium hydroxide, ethyl alcohol and all the other chemicals were analytical-grade reagents and purchased form Sinopharm Chemical Reagent Co. LTD. (Shanghai, China).

A buffalo rat liver (BRL) cell line obtained from the Cell Bank of Type Culture Collection at the Chinese Academy of Sciences (Shanghai, China). BRL cells were cultured in high-glucose DMEM medium supplemented with 10% fetal serum (FBS, Gibco), 100 U/mL penicillin and 100 ug/mL streptomycin at 37 °C with an atmosphere of 5% CO_2_.

Male ICR mice (20 ± 1 g) and male SD rats (250 ± 10 g) were purchased from SLACCAS Laboratory Animal Co, Ltd. (Shanghai, China). All animal experimental procedures were approved by the Independent Ethics Committee of Shanghai Ninth People’s Hospital, Shanghai JiaoTong University School of Medicine. All experiments followed were conducted in accordance with Division of Laboratory Animal Medicine guidelines.

### Preparation of radiolabeled and non-radioactive MBG nanospheres

A sol-gel reaction in combination the hydrothermal method was applied to prepare radiolabeled MBG nanospheres. Briefly, 0.46 g NaOH was dissolved in 120 mL ddH_2_O to form the alkaline solution, and 1.0 g of PVP was added and stirred for 10 minutes. 1.4 g CTAB as structure inducer was dissolved to the solution and mixed for 60 minutes. Then, Ca(NO_3_)_2_·4H_2_O and calcium-45 radionuclide (74 MBq/batch) were pre-mixed, and TEOS, TEP and were added subsequently with continuous stirring for 24 h. The molar ratio of Ca: P: Si is 15: 5: 80. The solution were collected and washed by ddH_2_O for 3 times, and then sealed in Teflon-lined autoclaves at 80 °C for 48 h. Finally, the products were dried at 80 °C for 12 h and calcined at 550 °C for 5 h to obtain radiolabeled MBG nanospheres (^45^Ca-MBG nanospheres). Non-radioactive MBG nanospheres were synthesized in the same procedure without adding calcium-45 radionuclide.

### Modification of MBG nanospheres

The resulting ^45^Ca-MBG and non-radioactive MBG nanospheres were modified as necessary with the APTES. APTES in ethyl alcohol [2% (v%)] was stirred for 1 h at room temperature. Thereafter, MBG nanospheres were added at a concentration of 10% and incubated with stirring for 6 h at 60 °C under the reflux condensation. After washing with alcohol for three times and vacuum drying, the modified ^45^Ca-MBG and non-radioactive MBG nanospheres were collected respectively.

### Fluorescent labeling of MBG nanospheres

Ten milligrams of modified non-radioactive MBG nanospheres were dissolved in 10 mL of ethyl alcohol. 1 mM Cy-7 NHS was added in 0.1 mL DMSO to form a dye stock solution. While stirring the MBG nanospheres solution, 100 μL dye stock solution was added and incubated at room temperature for 4 h. The Cy7 labeled MBG nanospheres (Cy7-MBG nanospheres) were finally obtained through centrifuging and resuspending for repeating 3 times.

### Characterization of MBG nanospheres

The surface morphology, inner microstructure and element composition characteristic bands of the MBG nanospheres were analyzed by scanning electron microscopy (SEM, JSM-6700F, JEOL), transmission electron microscopy (TEM, JEM-2010, JEOL), and energy-dispersive spectrometer (EDS, EDAX, AMETEK). Fourier transform infrared spectroscopy (FTIR, EQUINOX 55, Bruker Co) was performed to analyze the surface characteristics of nanospheres with a scan range of 400–4000 cm^−1^. Lastly, the characterization of size and zeta potential of the MBG nanospheres in physiological saline were performed on a Zetasizer Nano ZSP (Malvern Instruments Ltd.).

### Identification of ^45^Ca-MBG nanospheres

The ^45^Ca-MBG nanospheres were dispersed in ethyl alcohol at the concentration of 1 mg/mL, and 2 μL ^45^Ca-MBG nanospheres solution was extracted and subjected to paper chromatography using instant thin layer chromatography-silica gel (ITLC-SG) for 20 minutes at room temperature, with physiological saline as the solvent. The dilute calcium-45 radionuclide set as control. The radioactive substance was identified by Tri-Carb 3110TR Liquid Scintillation Counters (Perkin Elmer, USA). According to the proportion of the origin position’s radioactivity in ITLC-SG, the purity of ^45^Ca-MBG nanospheres was estimated.

### Biodegradation of MBG nanospheres *in vitro*

The biodegradation of MBG nanospheres was measured according to Wang and Chen’s researches^24^,^34^. MBG nanospheres (10 mg for each) was dispersed into 1 mL modified simulated body fluid (mSBF)^35^ and transferred to dialysis tube (molecular weight cut-off is 10 K), then the dialysis tube was immersed into 39 mL mSBF in a polypropylene container and shook continuously at a rate of 150 rpm at 37 °C. 5 mL supernatant was extracted at each time point (1h, 1d, 3d, 5d and 7d) and an equal volume of fresh mSBF was added. The concentrations of silicon in the supernatants were determined using inductively coupled plasma optical emission spectroscopy (ICP-OES, Agilent, USA). The released silicon concentrations were determined by the following equation:


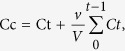


where Cc is the corrected concentration of free silicon at time t, Ct is the apparent concentration obtained by sampling at time t, v is the volume of sample taken (5 mL), and V is the total volume of immersion fluid (40 mL).

The total silicon percentage of MBG nanospheres was measured as follows: 10 mg MBG was poured into 4 mL HNO_3_ solution and used a microwave digester. Then, the total free silicon concentration was measured with ICP-OES.

### Quantitative radionuclide tracing of ^45^Ca-MBG nanospheres

Twenty five mice were divided randomly into five groups according to the presumed measurement time (1d, 3d, 7d, 15d and 30d). The concentration and injection dose of ^45^Ca-MBG nanospheres were 1 mg/mL in physiological saline and 10 mg/kg body weight, respectively. The mice were injected intravenously via tail vein with ^45^Ca-MBG nanospheres. The blood, heart, lungs, liver, spleen, kidneys, stomach and intestine were harvested and weighed after each time point post-injection. The collected tissue and organs were dissolved with 1 mL hydrogen peroxide and 1 mL perchloric acid in a 60 °C water bath for 2 h. Then, each 200 μL aliquot of the dissolved samples was mixed with 4 mL of Hionic-Fluor liquid scintillation cocktail (Ultima Gold™), and the mixture was shaken for 60 s and protected from light for 2 h. After the linear correction of standard ^45^Ca, the radioactivity of the samples was counted for 1 min to detect the tissue radioactivity by Tri-Carb 3110TR Liquid Scintillation Counters (Perkin Elmer, USA). The tissue distribution was expressed as the percentage injected dose per gram tissue (%ID/g).

### Qualitative tracing of Cy7-MBG nanospheres

Mice were injected intravenously by tail vein with Cy7-MBG nanospheres at the concentration of 1 mg/ml and a dose of 10 mg/kg. NIR flurescence images of the mice were recorded at the presumed measurement time using an imaging system (Berthold Technologies Co., Germany). Five anesthetized animals at a time were recorded for *in vivo* real-time imaging, and then sacrificed to harvest the major organs including heart, lung, liver, spleen, kidney and intestine. After washing with normal saline, tissues were subject to fluorescent imaging. The animals and tissues were recorded with selected excitation and emission band-pass filter and exposed for 0.1 s

### TEM observation and EDS analysis of MBG nanospheres in liver

Mice were injected intravenously with MBG nanospheres at a dose above-mentioned and sacrificed at 7 and 30 days. The liver was harvested and immersion-fixed in 2% glutaraldehyde for 2 h at 4 °C. Tissue sections were prepared after rinse, dehydration and embedded in ethoxyline resin. The sections were processed for evaluation the subcellular distribution and elementary composition of MBG nanospheres in liver by TEM-EDS (JEM-1400, JEOL).

### Cell viability assays

BRL cell viability was determined using a Cell Counting Kit-8 assay (CCK-8; Beyotime Biotech Ltd). BRL cell cultures were prepared at approximately 10,000 cells per well in 96-well plates. After 24 hours, the serial dilutions of MBG nanospheres (25, 50, 100, 200 and 400 μg/mL) were added, and the cultures were incubated for 24 h. A volume of ten microlitre of CCK-8 was then added to each well, and the plates were incubated for 4 h at 37 °C. The absorbance of formazan was measured at 450 nm using a microplate reader (Multiskan™ GO, Thermo Scientific, USA).

### Cellular uptake of ^45^Ca-MBG nanospheres

BRL cells were seeded in six-well plates at 4 × 10^5^ cells per well and cultured 24 h. Then the cells were treated with ^45^Ca-MBG nanospheres at 100 μg/mL for 1, 3, 6, 12 and 24 h. Subsequently, cells were washed three times with PBS, trypsinized and collected. The digested cells pellets was blended with 2 mL of Hionic-Fluor liquid scintillation cocktail (Ultima Gold™), and the radioactivity of the mixture was measured by liquid scintillation counting (LSC).

### Subcellular location of MBG nanospheres

BRL cells were incubated with 100 ug/mL ^45^Ca-MBG, and collected at 1, 3, 6, 12 and 24 h after the treatment. Cells were washed three times with PBS and homogenized with optimal gentle strokes. After homogenization, the crude nuclear fraction, the crude mitochondrial fraction and other cytoplasmic fraction were separated by differential centrifugation and Mitochondria/Nuclei Isolation Kit (KeyGEN BioTECH). The each fraction was digested, and 100 μL aliquot of the digested samples was mixed with 2 mL scintillation cocktail, and radioactivity in each fraction was measured.

To further observe the localization of MBG nanospnheres, BRL cells were exposed to nanospheres for 24 h and analyzed by TEM (CM-120, PHILIP, Netherlands). Briefly, BRL cells were seeded in a dish. After incubation for 24 h with nanospheres (100 μg/mL), the cells were washed with PBS solution, pelleted and fixed in PBS solution containing 2% glutaraldehyde, dehydrated through and embedded in epoxy resin. Thin sections containing the cells were placed on the grids and stained with lead citrate, and imaged under TEM.

### Intracellular ROS measurement

The production of intracellular reactive oxygen species (ROS) was measured by performing flow cytometry using DCFH-DA (Applygen, Beijing, China). Briefly, a DCFH-DA stock solution (in methanol) was diluted 2000-fold in cell culture medium without serum to yield a 5 mM working solution. After the exposure of BRL to MBG nanospheres (100 μg/mL) for 24 h, the cells in 6-well plates were washed twice with PBS and incubated in 1 mL working solution of DCFH-DA at 37 °C in dark for 30 minutes. Then the cells were washed with cold PBS and resuspended in the PBS for analysis of intracellular ROS by a flow cytometer (Guava easyCyte, Millipore, USA). The data were normalized to mean fluorescence intensity (MFI) values of the control cells.

### Excretory assay of ^45^Ca-MBG nanospheres

Five SD rats were injected intravenously via tail vein with ^45^Ca-MBG nanospheres at the concentration of 1 mg/ml and a dose of 10 mg/kg. Urine and feces of rats, which was collected daily using metabolic cages for 30 days, were weighted. After digested with perchloric acid and hydrogen peroxide in a 60 °C water bath for 2 h, 200 μL aliquot of the dissolved samples was blended with 4 mL liquid scintillation cocktail and counted. The excretion of ^45^Ca-MBG nanospheres was evaluated in the form of excretory rate per day (excretory rate/d) and accumulated excretory rate expressed as the percentage injected dose (%ID).

### Assessment of systemic toxicity

MBG nanospheres suspensions in physiological saline (1 mg/mL) were injected intravenously into ten mice through tail vein at 10 mg/kg for each day. Intravenous injections of sterilized physiological saline were given to mice (n = 10) as control. After 7 days of continuous injection, blood was drawn to measure the following clinical biochemical parameters: blood glucose (GLU), seralbumin (ALB), alanine aminotransferase (ALT), aspartate aminotransferase (AST), alkaline phosphatase (ALP), total bile acid (TBIL), blood urea nitrogen (BUN), uric acid (UA), creatinine (CREA) and cholesterol (CHOL). Then mice were sacrificed the organs, including heart, lung, liver, spleen, kidneys, stomach and intestines, were harvested and immediately fixed in a 10% formalin solution. Histopathology tests were performed using standard laboratory procedures. After hematoxylin-eosin staining, the histopathological reaction of major organs was evaluated using an optical microscope.

### Statistical analysis

Data were expressed as the mean ± standard deviation. Statistical analyses were performed using SPSS software (v 20.0; IBM Corporation, Armonk, NY, USA), and statistical comparisons were analyzed using the Student’s t-test. The differences were considered to be significant when the p value was less than 0.05.

## Additional Information

**How to cite this article**: Sui, B. *et al*. Drug-loadable Mesoporous Bioactive Glass Nanospheres: Biodistribution, Clearance, BRL Cellular Location and Systemic Risk Assessment via ^45^Ca Labelling and Histological Analysis. *Sci. Rep.* **6**, 33443; doi: 10.1038/srep33443 (2016).

## Supplementary Material

Supplementary Information
