# Supplementary material for: Drug-loadable Mesoporous Bioactive Glass Nanospheres: Biodistribution, Clearance, BRL Cellular Location and Systemic Risk Assessment via 45Ca Labelling and Histological Analysis
Source: Sci Rep. 2016 Sep 15;6:33443. doi: 10.1038/srep33443 (PMC5024120; doi:10.1038/srep33443)
Supplement: Supplementary Information [file srep33443-s1.pdf]

Drug-loadable Mesoporous Bioactive Glass Nanospheres: Biodistribution, Clearance, BRL Cellular Location and Systemic Risk Assessment via ^{45}Ca Labelling and Histological Analysis

Baiyan Sui [1], Gaoren Zhong[2] and Jiao Sun[1,*]

[1] Shanghai Biomaterials Research & Testing Center, Shanghai Key Laboratory of Stomatology, Ninth People's Hospital, Shanghai Jiaotong University School of Medicine, Shanghai 200023, China

[2] School of Pharmacy, Fudan University, Shanghai 201203, China

*Corresponding Author: E-mail: jiaosun59@126.com

Address: No. 427, Ju-men Road, Shanghai 200023, China.

Tel.:+86 21 63034903; Fax: +86 21 63011643.

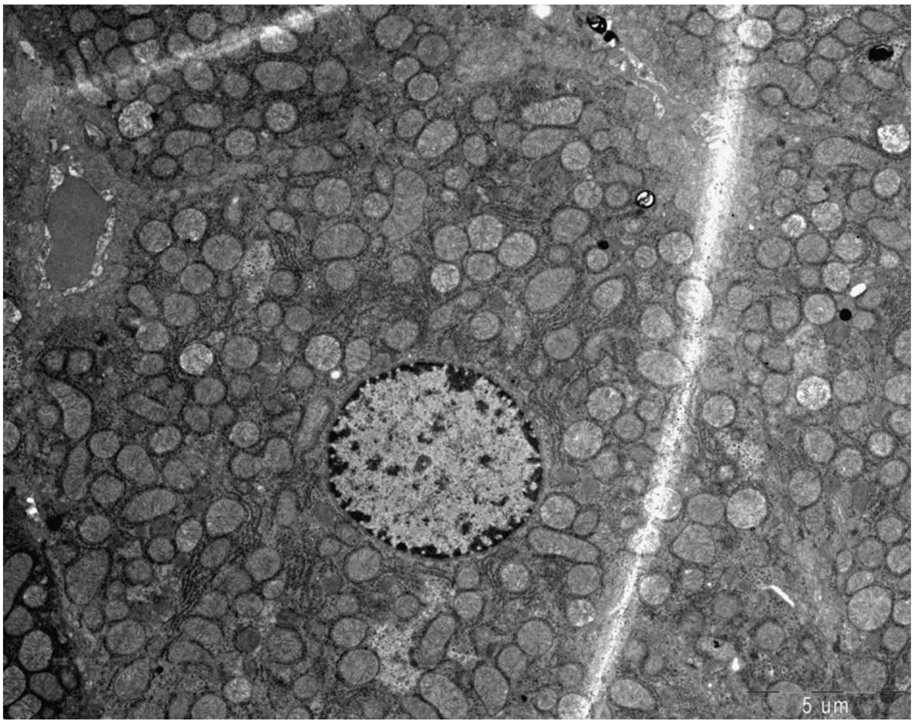

Figure.1 TEM images analysis of liver after injection with MBG nanospheres at 30 d.

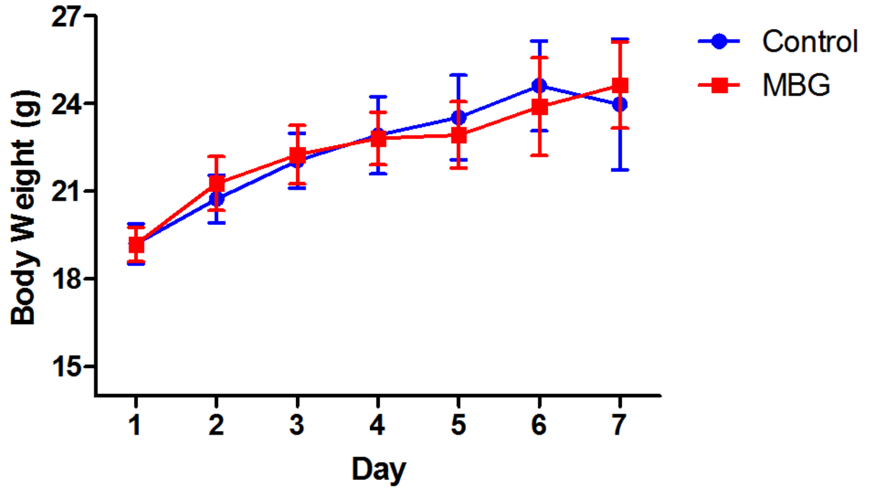

Figure.2 Average body weights of mice injected (i.v.) with MBG nanospheres once per day for 7 days. The results are presented as mean values ±SD.

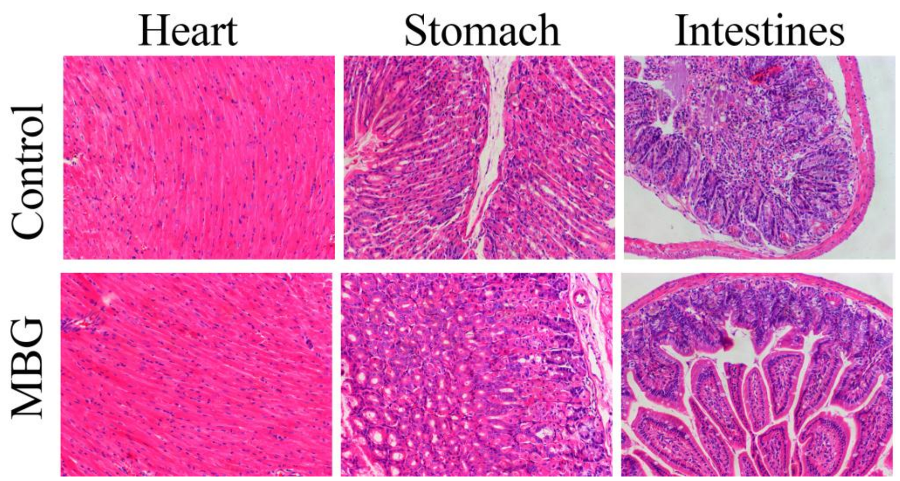

Figure.3 Sections from mice tissues after continuous injection with MBG nanospheres. All sections were stained with H&E and observed under a light microscope at 200× magnification.
